# Adaptable 2D to 3D Stereo Vision Image Conversion Based on a Deep Convolutional Neural Network and Fast Inpaint Algorithm

**DOI:** 10.3390/e25081212

**Published:** 2023-08-15

**Authors:** Tomasz Hachaj

**Affiliations:** Faculty of Electrical Engineering, Automatics, Computer Science and Biomedical Engineering, AGH University of Krakow, Al. Mickiewicza 30, 30-059 Krakow, Poland; thachaj@agh.edu.pl

**Keywords:** monocular stereo reconstruction, 2D to 3D, stereoscopy, depth, disparity, convolutional neural network, depth image based rendering, DIBR

## Abstract

Algorithms for converting 2D to 3D are gaining importance following the hiatus brought about by the discontinuation of 3D TV production; this is due to the high availability and popularity of virtual reality systems that use stereo vision. In this paper, several depth image-based rendering (DIBR) approaches using state-of-the-art single-frame depth generation neural networks and inpaint algorithms are proposed and validated, including a novel very fast inpaint (FAST). FAST significantly exceeds the speed of currently used inpaint algorithms by reducing computational complexity, without degrading the quality of the resulting image. The role of the inpaint algorithm is to fill in missing pixels in the stereo pair estimated by DIBR. Missing estimated pixels appear at the boundaries of areas that differ significantly in their estimated distance from the observer. In addition, we propose parameterizing DIBR using a singular, easy-to-interpret adaptable parameter that can be adjusted online according to the preferences of the user who views the visualization. This single parameter governs both the camera parameters and the maximum binocular disparity. The proposed solutions are also compared with a fully automatic 2D to 3D mapping solution. The algorithm proposed in this work, which features intuitive disparity steering, the foundational deep neural network MiDaS, and the FAST inpaint algorithm, received considerable acclaim from evaluators. The mean absolute error of the proposed solution does not contain statistically significant differences from state-of-the-art approaches like Deep3D and other DIBR-based approaches using different inpaint functions. Since both the source codes and the generated videos are available for download, all experiments can be reproduced, and one can apply our algorithm to any selected video or single image to convert it.

## 1. Introduction

Algorithms for converting 2D to 3D are gaining importance following the hiatus brought about by the discontinuation of 3D TV production; this is due to the high availability and popularity of virtual reality systems that use stereo vision. The majority of video content remains in 2D. Thus, to enjoy the full functionality of stereo vision systems, it is important to create solutions that allow fast, reliable, and adaptable 3D transformations. By ‘adaptable’, we refer to such approaches that can adapt the binocular disparity to the recording, the hardware on which it is displayed, and the user’s preferences. Since the user’s perception of 3D is subjective in practice, this requires, to some extent, real-time adaptability of disparity adjustments without the need to retrain a deep learning model.

Stereo vision is a very important technique used in the visualization of three-dimensional objects [1,2]. In order to use stereo vision to display 3D images, it is necessary to properly prepare a pair of images to be projected to the left and right eye of the observer. If we are dealing with a 2D film, which consists only of a single image (identical for both the left and right eye), it is necessary to transform this 2D image in such a way that a stereo vision image is formed from it.

One frequently used way to generate stereo images is to use depth image-based rendering (DIBR). DIBR involves performing distance estimation of individual objects that are visible in an image via a sequence of images or a single image. After the depth map is generated, it is used together with the input image to calculate the stereo pair [3,4,5,6,7,8,9].

Before deep learning became widespread for depth image estimation and stereo vision estimation, several methods were proposed that used other approaches. Among the non-deep learning methods are skeleton line/edge tracking [10,11], object segmentation [12,13], bilateral filtering [14,15], trilateral filtering [16], planar transformation between images [17], motion information between consecutive frames [18,19], the Welsch M-estimator [20], residual-driven optimization [21], and depth from motion/optical flow [5]. A survey on 2D to 3D video conversion can be found in [22].

Currently, the most commonly applied algorithms for distance estimation use deep learning techniques. These networks use well-known backbone architecture with U-net multi-level feature extractors [23]. Many methods use supervised training [24,25,26,27,28], while some use unsupervised neural network weight optimization [29,30]. Reviews on deep learning for monocular depth estimation can be found in references [31,32,33,34,35,36].

In recent years, 2D to 3D conversion methods using deep learning, without directly implementing DIBR, have also emerged. The solutions described in references [37,38] use deep neural networks that allow the direct generation of stereo pairs from a single RGB image as well as from a sequence of images. The network is directly trained using stereo pairs without ground truth depth data. Reference [9] describes a learning-based technique to automatically convert 2D panorama (static) images to stereoscopic versions. The authors in these works point out that obtaining a stereo pair that is useful to the observer, i.e., one that gives the impression of three-dimensionality and is comfortable to view (for example, does not cause headaches), requires analysis from the level of minimization of the loss function, e.g., mean absolute error, and subjective evaluations by 3D system users.

Noteworthy applications of 2D to 3D conversion algorithms include automatic generation of 3D environments to simplify 3D modeling processes [39], the creation of stereo vision movies to enhance immersion [8], and the 2D to 3D conversion for medical education purposes [40]). An overview of 2D to 3D conversion systems and their applications can be found in reference [41].

Based on the literature discussed above, it can be concluded that 2D to 3D image conversion is an open and complex topic in which a single (and universally applicable) solution has not been developed. There are two main groups of methods for that task: DIBR-based and direct image to stereo pair generation (for example, [37]). The DIBR-based approach is based on a mathematical model and allows the direct use of depth image generation algorithms, including single-frame methods that work on both single images and video sequences. The DIBR model also allows controlling stereo pair parameters related to the optics of the stereo vision system. Based on the literature reviewed, no work has yet been published that compares the use of different deep neural networks (DNNs) for single-frame image generation in 2D to stereo vision conversion. In this paper, several depth image-based rendering DIBR approaches using state-of-the-art single-frame depth generation neural networks and inpaint algorithms are proposed and validated, including a novel very fast inpaint (FAST). FAST significantly exceeds the speed of currently used inpaint algorithms by reducing computational complexity, without degrading the quality of the resulting image. In addition, we introduce a parameterization of DIBR using a single, easy-to-interpret parameter that can be adjusted online, according to the preferences of the user who views the visualization. The proposed solutions are also compared with automatic 2D to 3D mapping, namely Deep3D [37], which is a very popular state-of-the-art algorithm.

## 2. Materials and Methods

The most common assumption is that a 2D image, from which a stereoscopic image pair is generated, is representative of the left camera image (left image), and our task is to estimate the right camera image (right image). DIBR algorithms use depth images, also called depth maps, which are also estimated from the left image. In this section, the algorithm proposed in this work will be described, which allows fast and adaptive generation of stereo vision image pairs.

### 2.1. Depth Image Estimation

Currently, the most common approach to depth image generation is to use a suitable deep neural network. Researchers of various solutions focus on improving accuracy and achieving relatively fast image processing speeds. To test the effectiveness of the solution in this work, we selected five architectures from among the available depth prediction DNNs. These networks were chosen because they are based on different architectures of depth feature extractors and are relatively new solutions that have gained popularity in various applications. The dense depth (DD) network uses a pre-trained DenseNet [42] and a U-Net architecture for multi-scale depth image filtering and reconstruction. The network implementation is available at https://github.com/ialhashim/DenseDepth (accessed on 10 June 2023). The dense depth small (DD-S) network has a similar architecture to DD, but by reducing the number of layers that reconstruct the output signal, a faster network speed is achieved. The network’s implementation is available at https://github.com/browarsoftware/tello_obstacles (accessed on 10 June 2023). The solution [27] (MiDaS) uses ResNet [43] and multi-scaled U-Net. Ref. [27] presents three versions: small (MiDaS-S), hybrid (MiDaS-H), and large (MiDaS-L); these variations differ by their operational speed and accuracy of distance estimation. The network’s implementation is available at https://github.com/isl-org/MiDaS (accessed on 10 June 2023).

As DD is based on the relatively simple DenseNet-169 backbone, which consists of forward connections without residuals, it is possible to resize the decoder by forming connections with the chosen resolution via skip connections. The biggest advantage of DD is that—with the DenseNet backbone architecture—one can create deep estimation networks that significantly vary in the number of weights and processing speed. The disadvantage, on the other hand, is the lack of residuals in the backbone, which somewhat limits the expressive properties of the network. The DD and DD-S networks exploit the DenseNet-169 backbone, but DD has one more extractor than DD-S. DD, therefore, performs slower than DD-S but is more precise. Networks in the MiDaS family use ResNet as the backbone. The MiDaS-S, MiDaS-H, and MiDaS-L models differ in the size of the ResNet and, thus, in speed and accuracy. The more layers there are in the ResNet, the better depth estimation it offers in exchange for the speed of operation, which decreases as the number of layers increases.

### 2.2. Depth Image-Based Rendering

If we have a depth image and a left image, we can use them to generate the right image from the stereo pair using a depth image-based rendering (DIBR) principle [3,44]. According to this approach, the amount of the sensor shift, *h*, is defined with the following equations:

(1) $$h=-t_{x}\frac{f}{Z}$$

and

(2) $$t_{x}=\left\{\begin{array}{ccc} -t_{c} & :left-eye & view\\ t_{c} & :right-eye & view \end{array}\right.$$

where $t_{c}$ is interaxial distance, *f* is the focal length, and *Z* is the convergence distance (depth value of current pixel).

**Figure 1:**
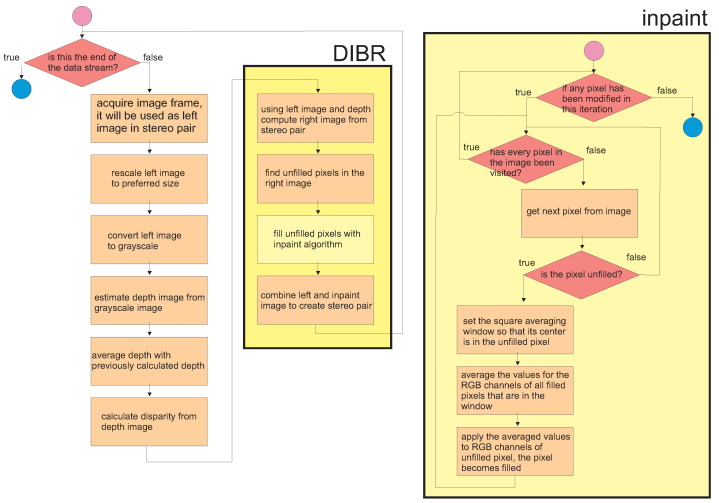
Flowcharts of Algorithms 1 and 2.

The transformation (1) is performed on all pixels in the left and right images. Since it is a per-pixel operation, in areas with high disparity, characteristic “holes” are created, the values of which must be filled by interpolating values from neighboring areas where the color is known. A direct application of the approach in (1) degrades both the right and left stereo vision pair images. That is why some approaches, e.g., ref. [6], only perform a transformation of one of the images, leaving the other unchanged. To apply the approach used in (1), one needs to specify parameters $t_{c}$ and *f*, which can vary, depending on the video signal one is dealing with. The stereo vision system one uses may also depend on the preference of the viewer. Taking this into account, we can simplify the above equations as follows:

(3) h=−D·MaxDisp

where *D* is a depth image with values in the range of [0,1], and *MaxDisp* is the maximal disparity between the left and right images. With this approach, it is possible to control the depth of the image generated with DIBR by using a single parameter. This approach (using parametrized maximal disparity) is presented in pseudo-code form in Algorithm 1. In Figure 1, flowcharts of Algorithms 1 and 2 are presented.
**Algorithm 1:** Depth image-based rendering (DIBR) algorithm with parameterized maximal disparity**Inputs**:*MaxDisp*—maximal disparity in output image, *θ*—averaging coefficient, h,w—height and width of the image, inpaintAlg—inpaint algorithm that fills holes inthe DIBR image, model—DNN (or any other) model to estimate the depth imagefrom the grayscale image.**Outputs**:Algorithm continuously estimates the right stereo image and stores it in variablerightImgInpaint
// initialize previous image as empty.
depthPrev ← ⌀
// algorithm runs continuously
**while**
*true*
**do**
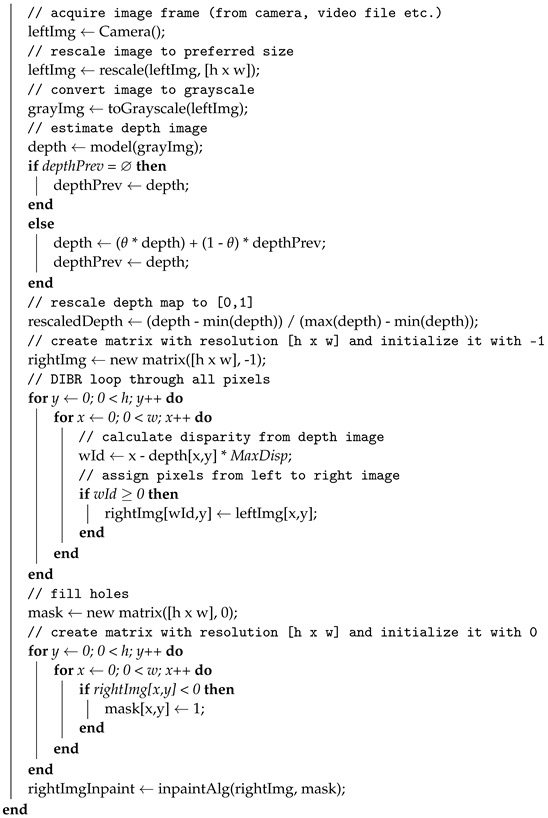


**Algorithm 2:** Fast algorithm for image inpaint (FAST)**Inputs**:Image—input image in which holes will be filled, mask—matrix where 1indicates pixels that should be filled, h, w—height and width of the image,windowSize—size of the averaging window.**Outputs**: Image with filled holes.
// initialize previous image as empty
depthPrev ← ⌀
// algorithm runs until all holes are filled
change ← True
**while**
*change*
**do**


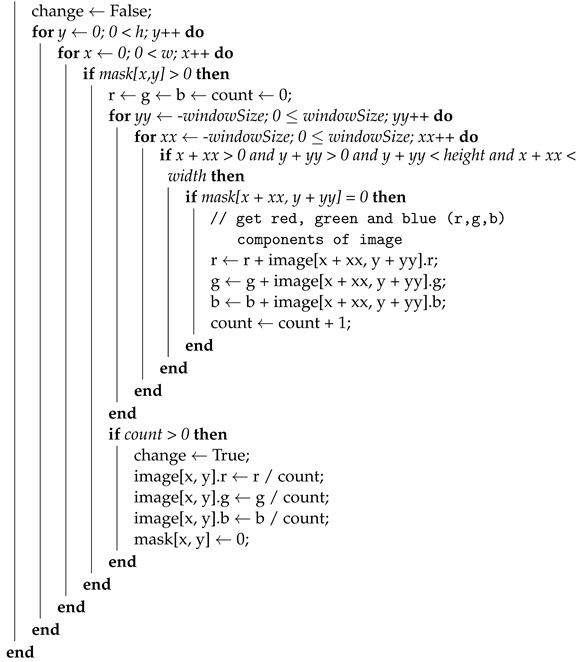



### 2.3. Fast Inpaint Algorithm

The right image generated by the DIBR algorithm contains inaccuracies (or holes), which are caused by the fact that pixels from the right image are shifted to the left at the border of the high- and low-disparity areas. Since the left camera does not register the colors of the pixels in these areas, these holes must be filled in. For this, one can use algorithms from the digital inpainting group, which enable the reconstruction of small damaged portions of an image. These algorithms use the input image and a binary map of areas as input parameters, where the pixels whose values should be calculated are indicated. Very popular digital inpainting algorithms include the technique based on the fast marching method [45] (Telea algorithm) and the Navier–Stokes-based approach described in [46]. The above methods are gradient-based approaches, which have high efficiency even over extensive (relatively wide and high) areas of holes. In practice, however, the holes generated by DIBR tend to be vertical lines of relatively narrow widths. For this reason, in most cases, it is not necessary to find narrow-band areas to be filled by the estimated pixels. Based on this observation, in this work, a much faster approach than the ones used in [45,46] is proposed, which uses the fast estimation of pixels inside holes by supplementing them with average values of the surrounding pixels that are not holes. The algorithm iteratively walks through all the holes that are stored in the mask image as nonzero pixels until all holes are filled. For even a small averaging window size (windowSize parameter), a single iteration of the while loop is enough to fill all holes. The pseudo-code of the proposed algorithm (FAST inpaint) is presented in Algorithm 2.

**Figure 2:**
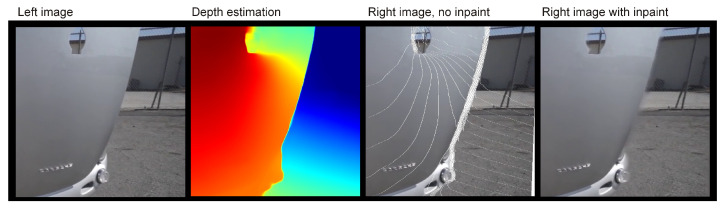
This figure visualizes the image processing results of Algorithms 1 and 2. From the left, one can see the input (**left**) image, the depth map estimated from it, the right image calculated with DIBR (white linear areas with unfilled pixels are clearly visible), and the **right** (result) image without holes, filled with the inpaint algorithm.

Figure 2 shows the image processing results of Algorithms 1 and 2. Before applying the inpaint algorithm, there are visible areas with white pixels where the DIBR method did not estimate the pixel color. These are areas located at the edges of surfaces, which differ substantially in their estimated distances from the observers, such as the area at the edge of a car’s trunk.

## 3. Results

The methods described in Section 2 were implemented using Python 3.10 via the following deep learning packages: TensorFlow 2.10, Keras 2.10, and Torch 1.11. For image processing, OpenCV–Python 4.7 was used; for speed-up calculations, a high-performance Python compiler, Numba 1.23, was utilized. All source code and data described in this article can be downloaded at https://github.com/browarsoftware/stereofast (accessed on 16 June 2023). The evaluation was performed on a PC equipped with an Intel i7-9700 3 GHz, 64 GB RAM, and an NVIDIA GeForce RTX 2060 GPU on Windows 10 OS. The GPU support was enabled.

In order to test Algorithms 1 and 2, which are proposed in this work, we conducted an experiment involving a series of tests.

- Quantitative study—using the stereo evaluation set KITTI [47,48]. The KITTI dataset consists of 200 test scenes that were recorded by a moving car via a stereo camera. The images show ordinary traffic involving cars. Static objects are visible, such as trees, road signs, etc. The original resolution of the data is 1242 × 375. The mean absolute error (MAE) was calculated between the right image and the estimation of the right image generated from the left image. We measured the speeds of the various algorithms, defined as the average processing time of the standard deviation of the animation frame, *pm*, and the number of frames per second (FPS).
- We conducted a qualitative study of the user experiences on the generated stereo videos based on the methodology from [9,49]. From 23 free recordings via Pixabay https://pixabay.com/ (accessed on 10 June 2023), stereo vision videos were generated, and five adults were asked to evaluate the quality of generated videos. Those people were university students, but they were not experts in the computer vision field. They also declared that they had no previous experience with viewing stereo vision videos using virtual reality systems, such as Oculus. Participants in the experiment watched the stereo vision videos using the VR system Oculus Quest 2 via the DeoVR QUEST app. In the DeoVR QUEST app, there is an option to turn off the stereo vision effect (the “Force mono projection on this video” option); test subjects were free to use it to see if the 3D impression was merely a suggestion. Each subject was able to replay a single recording many times.

### 3.1. Quantitative Study

Algorithm 1 (DIBR) was run using backbone networks [25] (DD), [26] (DD-S), and [27] (MiDaS-S, MiDaS-H, and MiDaS-L). The inpaint algorithms used were [46] (NS), [45] (Telea), and the FAST algorithm proposed in this work (the window size was set to 3). The maximal disparity (*MaxDisp*) parameters were set to 25, 20, and 75. The results were also compared with the Deep3D algorithm [37], which are available at https://github.com/HypoX64/Deep3D (accessed on 10 June 2023). Recordings from the KITTI dataset were processed in 640 × 360 and 1280 × 720 resolution. Regarding the DD backbone, depth image generation in 640 × 360 resolution was not possible due to the too-small size of the U-Net bottleneck. Quantitative evaluation results for the KITTI dataset with a resolution of 640 × 360 are presented in Table 1; results with a resolution of 1280 × 720 are presented in Table 2.

**Figure 3:**
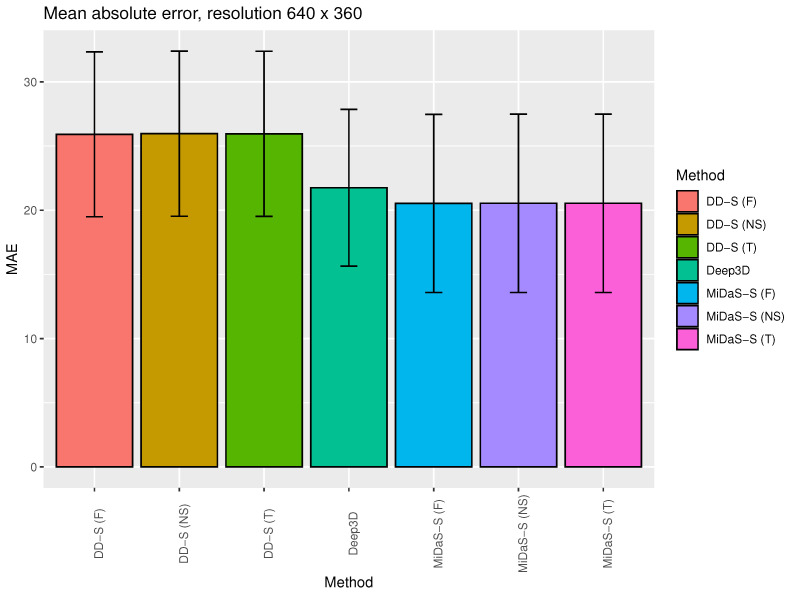
The average MAEs for the selected algorithms for 2D to 3D (stereo) generation with standard deviation bars (maximal disparity = 25). This is a visualization of the selected data presented in Table 1.

**Figure 4:**
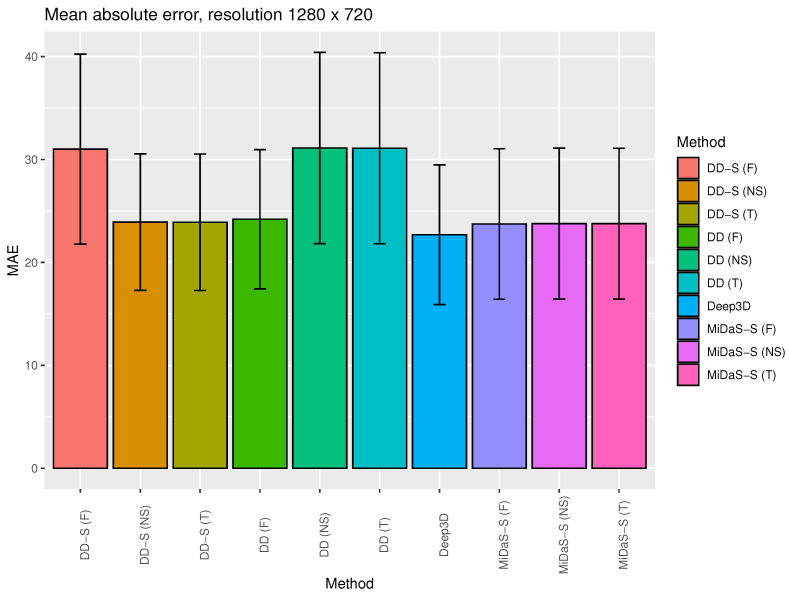
The average MAEs for the selected algorithms for 2D to 3D (stereo) generation with standard deviation bars (maximal disparity = 25). This is a visualization of the selected data presented in Table 2.

Figure 3 and Figure 4 show the average MAE value for selected 2D to 3D (stereo) generation algorithms with standard deviation bars (maximal disparity = 25). This is a visualization of selected data presented in Table 1 and Table 2.

### 3.2. Qualitative (User) Study

**Figure 5:**
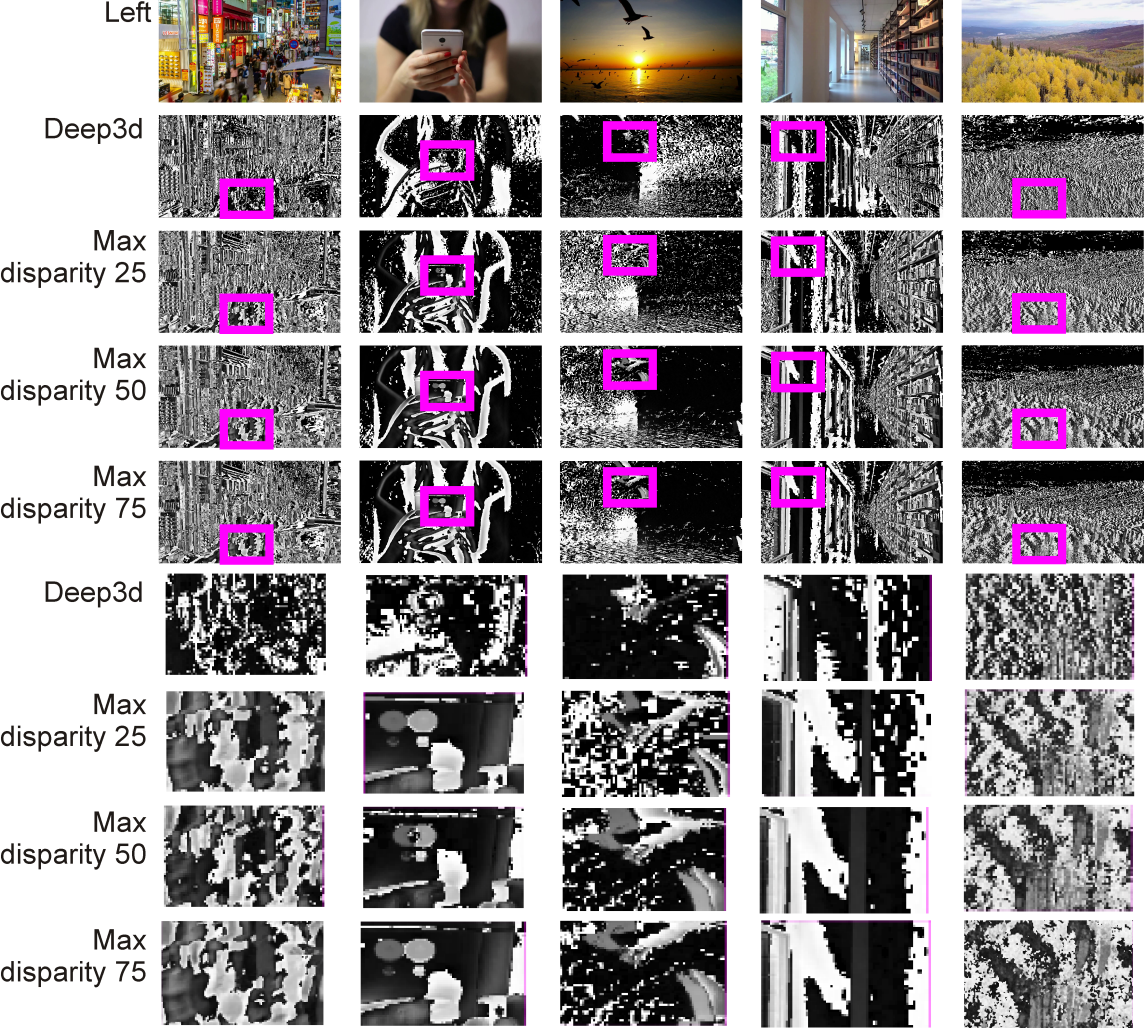
Example visualizations of stereo pairs generated by the Deep3D algorithm [37] and DIBR with the MiDaS backbone set at theta=0.75. The FAST inpaint algorithm, with a window size of 3, was applied for maximal disparities of 25, 50, and 75. The square in magenta highlights the enlarged areas containing the detailed results of each algorithm. The figure shows the differences between the left image and the generated right image, calculated as the absolute value from the per-pixel difference between these images in grayscale.

As can be seen in Table 1 and Table 2, there is no statistically significant difference from an MAE perspective between the various 2D to 3D (stereo) generations, DNN backbones, and inpaint method algorithms tested. However, the algorithms differ in their speeds. For the above reasons, a very important characteristic of an algorithm that determines its suitability is the viewer’s experience with the quality of the generated stereo vision video. To evaluate this, 23 stereo vision recordings were generated and 5 adults were asked to evaluate their quality. The recordings varied: they showed crowded streets of Seoul, close-ups of individuals, animals, panoramas of landscapes, and vehicles. Comparisons were made between the performance of the Deep3D algorithm and the proposed DIBR with the MiDaS backbone set at θ=0.75. The FAST inpaint algorithm, with a window size of 3, was applied for maximal disparities of 25, 50, and 75, maintaining a resolution of 1280×720. Thus, there were 23×4=92 videos. Example visualizations are shown in Figure 5.

Each participant in the experiment watched each of the 92 videos in random order, rating them on a scale of 1 to 10 (according to whether they had a 3D experience and whether they felt comfortable watching the video). A rating of 10 meant complete approval. The average results are presented in Table 3. The Deep3D and DIBR algorithms based on MiDaS and FAST were used for the experiment because they had the fastest speeds at similar MAE values compared to other tested approaches.

**Figure 6:**
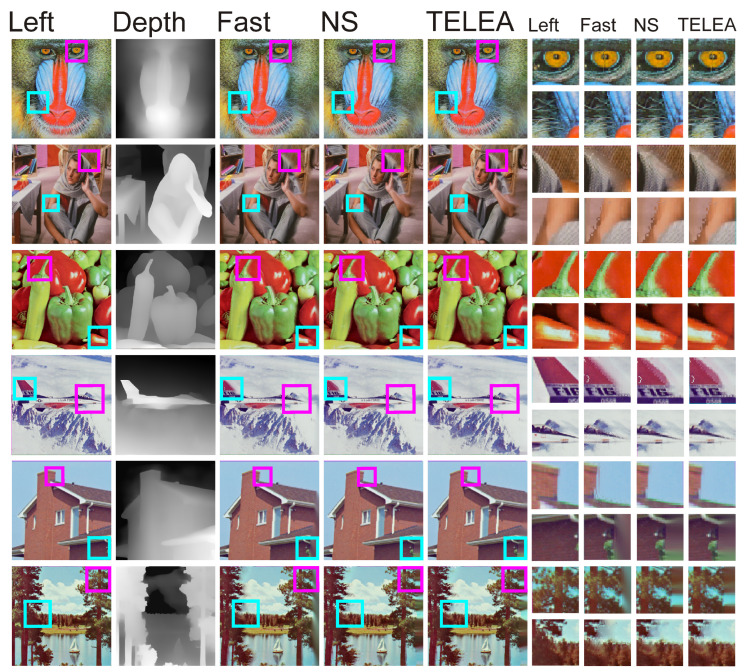
Comparison of the performances of inpaint methods NS, Telea, and FAST on the right image pairs. The magenta and cyan squares highlight the enlarged areas containing the detailed results of each algorithm.

Since the performances of the inpaint algorithms yielded virtually indistinguishable quantitative results on the right stereo vision image, a visual comparison was also made of the performances of the NS, Telea, and FAST methods on a well-known set of benchmark images. A visualization of this comparison is shown in Figure 6.

**Figure 7:**
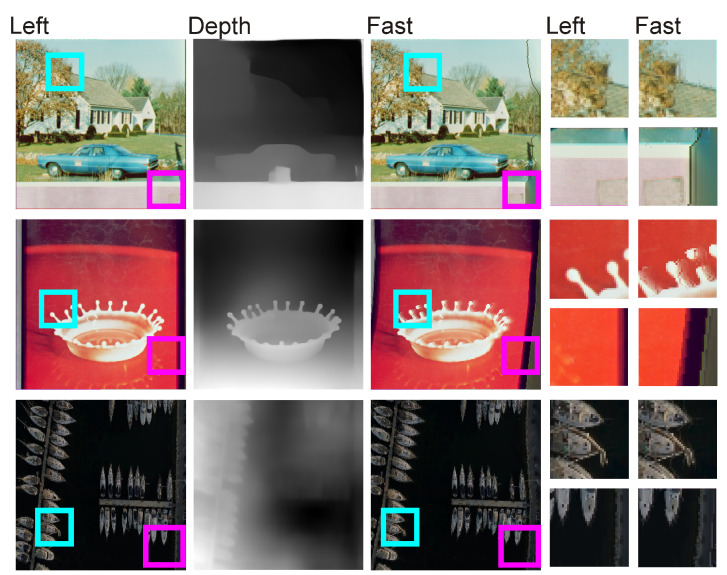
Visualization of the limitations of the proposed approach based on DIBR with MiDaS depth image estimation and FAST inpaint. The magenta and cyan square highlights the enlarged areas containing the detailed results of each algorithm.

The proposed approach, based on DIBR with DNN-based depth image estimation and FAST inpaint, has some limitations; examples are presented in Figure 7 and will be discussed in Section 4.

## 4. Discussion

According to the results presented in Table 1 and Table 2 and Figure 3 and Figure 4, there is no significant statistical difference between the tested algorithms when considering MAE. All algorithms returned similar results and had relatively high standard deviation values. The worst results were obtained for the DIBR method with the DD-S backbone. This is due to the fact that the DD-S network has lower depth image restoration efficiency than the other DNN algorithms that were tested (see the evaluation results in [26]). This leads to the intuitive conclusion that a very effective depth estimation algorithm is necessary for DIBR-type algorithms to be effective.

The algorithms differed significantly in their speed of operation. From the point of view of speed, there are two bottlenecks in DIBR-type algorithms: depth estimation and inpaint. Of the DNNs tested, the MiDaS-S algorithm was the fastest, followed by DD-S, MiDaS-H, MiDaS-L, and DD. Because the algorithms in the MiDaS group had a high accuracy of depth estimation compared to the others (see [27]), and because they ran faster than the other solutions tested, MiDaS was used in the qualitative (user) study. Regarding inpaint, the FAST algorithm proposed in this work ran several times faster than the NS or Telea algorithm—in the case of MiDaS-S with a resolution of 1280×720, it was nearly 4 times faster with a max disparity of 25, nearly 10 times faster with a max disparity of 50, and nearly 20 times faster with a max disparity of 75. FAST does not appear to be as affected in terms of speed reduction when increasing disparity when compared to NS and Telea. This is because FAST is not a gradient approach and does not use a narrow band. For this reason, the qualitative (user) study used FAST inpaint. The Deep3D algorithm showed the fastest performance; at a resolution of 640×360, it was nearly twice as fast as the DIBR with MiDaS-S and FAST inpaint; at a resolution of 1280×720, it was ∼1.6 faster than the DIBR with MiDaS-S and FAST inpaint. This algorithm was also used in the qualitative (user) study.

The qualitative study presented in Table 3 clearly indicates that DIBR-type algorithms better represent 3D. The Deep3D algorithm, when it comes to perceiving 3D, was rated very similar to DIBR, with a small maximum disparity. Participants judged that the stereo vision pair generated by Deep3D was, in many cases, too subtle and not “deep” enough to be treated as 3D at all. Increasing the max disparity also increased the 3D feel of the images. On the other hand, the increased disparity resulted in a decrease in viewing comfort, although not in direct proportion to the enhanced three-dimensionality. With the equation proposed in this work (3) users can conveniently modulate the maximum depth to their comfort. The lack of disparity adaptation for non-DIBR-based algorithms is a major drawback, as it does not allow adapting the received image to different video recordings, stereo vision systems, and the user’s preferences. Speed is not an advantage here; due to Deep3D’s low-perceived rating, it makes no sense to use this algorithm in practice. Examples showing the differences between the left animation frame and the generated right animation frame by individual images can be seen in the figure column. Regarding the library corridor (fourth column), all tested algorithms provided similar results; regarding the view of a tree-filled valley (fifth column), the Deep3D image is unfortunately relatively “flat” and lacks detail, including the distinctiveness of the trees in the foreground Figure 5; in the first column’s image (street of Seoul), as can be seen in the magnified window, Deep3D does not single out individual street participants as objects that are worth “highlighting”. The situations are also similar in the cases involving the cell phone video (second column) and birds (third column). In the library corridor case (fourth column), all tested algorithms presented similar results; regarding the view of a tree-filled valley (fifth column), the Deep3D image is—unfortunately—relatively “flat” and lacks detail; this also pertains to the clarity of the trees in the foreground.

Figure 6 shows a comparison of the performances of the three tested inpaint algorithms on well-known benchmark images. As one can see, none of the algorithms is able to escape some inaccuracies, which occur in particular at the borders of areas and differ in depth values. In the images of peppers, the airplane, and the roof, one can clearly see the irregularities that arise from the interpolation of pixels in the right image. We should note that each of the algorithms used generates almost identical inaccuracies, regardless of the inpaint methodology used. Inaccuracies in the form of vertical lines can also be seen in the eye of the mandrill. Another visible problem is the right edge of the image when the right edge of the depth image has a high value. Applying the DIBR results, in this case, results in a visible vertical line on the right image, where blurring due to pixel interpolation may be visible. Such phenomena, however, are practically impossible to eliminate in DIBR algorithms unless one decides to crop the right border.

Figure 7 shows, using the FAST algorithm as an example, special cases in which the proposed DIBR with MiDaS and the inpaint method will not return correct results. One such case occurs when there is a vertical line close to the edge of the image, such as in the image with the car and a house. For reasons described in the previous paragraph, the pixels of the right image are shifted to the left, which will also translate such a line to the left and make it visible in the right image. If the line is, for example, an artifact, as it is in the test image, it will be noticeable. The situation is analogous when generating a stereo pair for an image framed like the one showcasing a drop of water. The depth estimation algorithm can estimate that this framing is in the foreground and generate a visible distortion in the image. The last unfavorable situation is if the image is taken from a high altitude, so in practice, it is flat. Regarding convolutional neural network approaches, which essentially function as aggregated edge detectors, such images are interpreted as fragmented convex structures, despite the fact that the example image depicts yachts against a sea backdrop, photographed from a high altitude. Presently, such a situation cannot be prevented. However, despite these limitations, the algorithm proposed in this work creates images characterized by an impression of space, without fatiguing the eyes of the observer.

Modern depth image estimation algorithms overcome the limitations that earlier DIBR algorithms, which did not use DNNs, had. State-of-the-art single-frame depth estimation DNNs work very fast and allow direct application of the disparity-based model. This makes it possible to manually adapt the image while watching the video, which, in practice, is not possible with trained, out-of-the-box, and parameter-free models, like Deep3d. Among the tested solutions, the error expressed as MAE has large variability and is not statistically significant; thus, 3D visual perception and the comfort experienced while watching the movie are of the greatest importance. Moreover, single-image depth estimation methods allow calculating the depth of even a still image, which is a huge advantage over all methods based on optical image flow when estimating distance. The visible problem is “image float”, which can be partially eliminated by averaging adjacent frames (θ parameter in Algorithm 1).

The method proposed in this paper for generating stereo images is heuristic—there is no guarantee that it will find an accurate estimate of the missing stereo pair image. The DIBR algorithm itself, however, is not heuristic. The DIBR algorithm is based directly on the stereo camera model. From knowing the disparity between the left and right stereo pairs, the depth image can be estimated [50]. If the exact parameters of the stereo camera pair are known, the resulting depth image is precise and can be used to make accurate distance measurements, e.g., in tasks involving visual simultaneous localization and mapping (SLAM) [51]. Regarding the DIBR algorithms, we are dealing with the opposite task, i.e., with a known single image and a known distance map, we want to estimate an unknown image from a stereo pair. Usually, when we perform 2D to 3D conversion, the camera parameters are not known. That parameter can be precisely determined by the calibration process if the camera is available [52]. Because we usually do not have access to a camera’s parameters, a method that allows 2D to 3D conversion should be able to work on a variety of images, both static and video, without having to pre-estimate the parameters of the camera used for data acquisition. To ensure the successful operation of algorithms from the DIBR group, it is important to select an appropriate algorithm for estimating distance in images, as well as a method to supplement missing pixels that are usually present at the boundaries of areas that differ significantly in the distance from the observer. DIBR, although it is a method based on exact assumptions, allows finding an accurate solution that is limited by the accuracy of the algorithm estimating the distance and the algorithm supplementing the missing pixels in the resulting image.

Thanks to advancements in deep learning for single-frame depth estimation and the ever-increasing computational power of personal computers, it has become possible to implement complex algorithms that operate in real-time. It seems reasonable to update and extend the known DIBR algorithms with the latest distance estimation deep learning models and optimize inpaint algorithms in terms of their speed. Of course, deep learning algorithms that utilize convolutional networks that extract certain statistical properties of images in the training process are, in practice, heuristic. Similarly, inpaint algorithms, which usually analyze some local features of the image, do not guarantee finding optimal solutions. However, in image processing tasks, without full knowledge of the video sensors and the scene as a whole, where some elements may obscure others, one cannot expect to find an optimal solution but only an approximate one.

Another important issue to consider when designing the 2D to 3D conversion algorithm is how humans perceive stereo vision images. The most noticeable difference is the variability in human eye spacing, which means that virtually every person has a slightly different stereo vision system; images are perceived slightly differently in the right and left eyes. Modern hardware often provides adjustments for display positioning in the left and right eyes, but it does not automatically adapt the already rendered video.

The research underscores that understanding the quality of human viewer experiences while watching 3D movies is a complex and multidisciplinary problem that includes disciplines such as neuroscience [53,54,55]. Thus, it was necessary to test the proposed algorithm at different parameters of maximum disparity and estimate not only exact values, such as the MAE and algorithm speed, but also qualitative (user) opinions. Because of this, despite some apparent repeatability of the results, important conclusions can be drawn from Table 1, Table 2 and Table 3 regarding the effectiveness of the different configurations of the algorithm, depending on the depth estimation, DNN backbone, inpaint method, and a comparison with a fully automatic depth estimation approach, Deep3D. Thus, any algorithm that does not take into account complex issues, such as an individual’s biometric parameters or personal visual preferences, which are difficult to accurately estimate, will not work well for all the conditions. For this reason, regarding the method proposed in this work, great emphasis was placed on the fact that the algorithm’s user should be able to adjust the obtained stereo estimates to his/her preferences, to some extent. The natural choice was to choose parameters determining the maximum disparity between the left and right stereo pairs. The influence of the parameter is strictly determined by the DIBR equation and, thanks to the speed of the algorithm proposed in this work, it is possible to change this parameter while, for example, watching a movie. In order to speed up the whole solution, it was necessary to propose a fast inpaint algorithm, which, in addition to distance estimation, is a bottleneck of the DIBR-based approach. Although exact metrics are known to check the quality of the obtained solution from the area of image processing, in some special cases, when individual human perception is an important factor, it may be beneficial to have an evaluation conducted on a test group of individuals, as was the case in this work. Regarding the exact quality estimation methods for 2D to 3D conversion, MAE was used. The depth map estimation algorithms are the average relative error, root mean squared error, average ($log_{10}$) error, and threshold accuracy. Of course, these are only numerical values, which do not necessarily represent the usefulness of the results of these algorithms from the point of view of human perception. The algorithm proposed in this work does not require training as it directly uses trained neural networks to calculate the distance. Details of the training datasets and the training process can be found in the papers referenced in Section 2.1.

In conclusion, the proposed solution, thanks to the use of the latest depth estimation algorithms, a new fast inpaint algorithm, and the possibility of real-time adaptation of the maximum disparity, has the potential to become a useful and popular algorithm, serving both the scientific community and the commercial sector. This is made all the easier due to the fact that full source codes are included in the work.

## 5. Conclusions

In this work, a single-parameter 2D to 3D (stereo vision) conversion model was proposed. The algorithm was then compared with several available DIBR configurations and a parameter-free fully automatic 2D to 3D conversion model (Deep3D).

Based on the discussion presented in the previous section, we can conclude that observers have praised the DIBR algorithm proposed in this work—consisting of intuitive disparity steering based on Equation (3), backbone DNN MiDaS, and the FAST inpaint algorithm—as a method that generates steerable 3D on a variety of recordings with qualitative (user) scores that overcome state-of-the art fully automotive 2D to 3D conversion. The MAE of the proposed solution does not contain statistically significant differences from state-of-the-art approaches like Deep3D and DIBR based on other networks and state-of-the-art inpaint functions.

Despite some limitations that we discussed in the previous section, the accuracy, quality, and high speed of the proposed algorithm allows it to be applied to real-time applications and systems. Because depth estimation is not based on optical flow, since the algorithm uses single-frame depth estimation, this also makes the proposed method suitable for generating stereo vision 3D images from single animation frames. This is especially usable in video content where shots are characterized by static cameras, including computer games. Since both the source codes and the generated videos are available for download, all experiments can be reproduced; one can use the algorithm on any selected video or single image and convert it, for example, to be viewed via virtual reality glasses.

## Figures and Tables

**Table 1:** Quantitative evaluation results for the KITTI dataset with a resolution of 640 × 360.

Method	Backbone	MaxDisp.	Inpaint	MAE	Time	FPS
DIBR	MiDaS-S [27]	25	Fast	20.53±6.94	0.022±0.004	∼45
DIBR	MiDaS-S [27]	50	Fast	19.82±5.92	0.024±0.004	∼42
DIBR	MiDaS-S [27]	75	Fast	21.55±6.26	0.025±0.004	∼40
DIBR	MiDaS-S [27]	25	NS [46]	20.54±6.95	0.052±0.003	∼19
DIBR	MiDaS-S [27]	50	NS [46]	19.84±5.93	0.101±0.005	∼10
DIBR	MiDaS-S [27]	75	NS [46]	21.58±6.28	0.167±0.009	∼6
DIBR	MiDaS-S [27]	25	Telea [45]	20.54±6.95	0.082±0.006	∼12
DIBR	MiDaS-S [27]	50	Telea [45]	19.84±5.93	0.195±0.010	∼5
DIBR	MiDaS-S [27]	75	Telea [45]	21.57±6.28	0.339±0.015	∼3
DIBR	MiDaS-H [27]	25	Fast	19.67±6.84	0.144±0.005	∼7
DIBR	MiDaS-H [27]	50	Fast	19.12±5.77	0.143±0.004	∼7
DIBR	MiDaS-H [27]	75	Fast	21.35±6.52	0.145±0.006	∼7
DIBR	MiDaS-H [27]	25	NS [46]	19.64±6.85	0.162±0.003	∼6
DIBR	MiDaS-H [27]	50	NS [46]	19.11±5.77	0.223±0.006	∼4
DIBR	MiDaS-H [27]	75	NS [46]	21.38±6.57	0.261±0.004	∼4
DIBR	MiDaS-H [27]	25	Telea [45]	19.65±6.84	0.234±0.008	∼4
DIBR	MiDaS-H [27]	50	Telea [45]	19.11±5.77	0.343±0.010	∼3
DIBR	MiDaS-H [27]	75	Telea [45]	21.37±6.56	0.469±0.013	∼2
DIBR	MiDaS-L [27]	25	Fast	19.30±6.93	0.242±0.004	∼4
DIBR	MiDaS-L [27]	50	Fast	19.33±5.85	0.253±0.013	∼4
DIBR	MiDaS-L [27]	75	Fast	21.81±6.87	0.252±0.004	∼4
DIBR	MiDaS-L [27]	25	NS [46]	19.27±6.94	0.280±0.003	∼4
DIBR	MiDaS-L [27]	50	NS [46]	19.34±5.87	0.365±0.017	∼3
DIBR	MiDaS-L [27]	75	NS [46]	21.86±6.92	0.424±0.008	∼2
DIBR	MiDaS-L [27]	25	Telea [45]	19.28±6.94	0.352±0.005	∼3
DIBR	MiDaS-L [27]	50	Telea [45]	19.34±5.86	0.541±0.011	∼2
DIBR	MiDaS-L [27]	75	Telea [45]	21.85±6.91	0.768±0.020	∼1
DIBR	DD-S [26]	25	Fast	25.91±6.42	0.126±0.008	∼8
DIBR	DD-S [26]	50	Fast	28.25±7.12	0.138±0.013	∼7
DIBR	DD-S [26]	75	Fast	30.55±7.98	0.131±0.006	∼8
DIBR	DD-S [26]	25	NS [46]	25.96±6.43	0.172±0.024	∼6
DIBR	DD-S [26]	50	NS [46]	28.33±7.15	0.230±0.073	∼4
DIBR	DD-S [26]	75	NS [46]	30.65±8.02	0.443±0.122	∼2
DIBR	DD-S [26]	25	Telea [45]	25.95±6.43	0.316±0.048	∼3
DIBR	DD-S [26]	50	Telea [45]	28.31±7.14	0.665±0.111	∼9
DIBR	DD-S [26]	75	Telea [45]	30.62±8.01	0.990±0.219	∼1
Deep3D [37]	–	–	–	21.75±6.11	0.012±0.004	∼83

**Table 2:** Quantitative evaluation results for the KITTI dataset with a resolution of 1280 × 720.

Method	Backbone	MaxDisp.	Inpaint	MAE	Time [S]	FPS
DIBR	MiDaS-S [27]	25	Fast	23.73±7.31	0.053±0.003	∼19
DIBR	MiDaS-S [27]	50	Fast	23.14±7.35	0.053±0.001	∼19
DIBR	MiDaS-S [27]	75	Fast	22.31±7.46	0.057±0.007	∼18
DIBR	MiDaS-S [27]	25	NS [46]	23.77±7.34	0.199±0.010	∼5
DIBR	MiDaS-S [27]	50	NS [46]	23.18±7.38	0.609±0.026	∼2
DIBR	MiDaS-S [27]	75	NS [46]	22.34±7.49	1.155±0.046	∼1
DIBR	MiDaS-S [27]	25	Telea [45]	23.76±7.33	0.485±0.029	∼2
DIBR	MiDaS-S [27]	50	Telea [45]	23.17±7.37	1.541±0.099	<1
DIBR	MiDaS-S [27]	75	Telea [45]	22.33±7.48	2.528±0.130	<1
DIBR	MiDaS-H [27]	25	Fast	23.811±7.73	0.170±0.004	∼6
DIBR	MiDaS-H [27]	50	Fast	23.03±7.77	0.186±0.011	∼6
DIBR	MiDaS-H [27]	75	Fast	21.95±7.93	0.179±0.005	∼6
DIBR	MiDaS-H [27]	25	NS [46]	23.86±7.79	0.355±0.010	∼3
DIBR	MiDaS-H [27]	50	NS [46]	23.07±7.83	0.787±0.044	∼1
DIBR	MiDaS-H [27]	75	NS [46]	21.99±7.98	1.224±0.038	<1
DIBR	MiDaS-H [27]	25	Telea [45]	23.85±7.78	0.853±0.056	1
DIBR	MiDaS-H [27]	50	Telea [45]	23.07±7.82	1.944±0.059	<1
DIBR	MiDaS-H [27]	75	Telea [45]	21.99±7.97	3.094±0.120	<1
DIBR	MiDaS-L [27]	25	Fast	24.23±8.07	0.282±0.009	∼4
DIBR	MiDaS-L [27]	50	Fast	23.35±8.12	0.282±0.007	∼4
DIBR	MiDaS-L [27]	75	Fast	22.30±8.21	0.283±0.007	∼4
DIBR	MiDaS-L [27]	25	NS [46]	24.29±8.14	0.605±0.016	∼2
DIBR	MiDaS-L [27]	50	NS [46]	23.40±8.18	1.124±0.025	<1
DIBR	MiDaS-L [27]	75	NS [46]	22.35±8.26	1.742±0.059	<1
DIBR	MiDaS-L [27]	25	Telea [45]	24.29±8.13	1.176±0.036	<1
DIBR	MiDaS-L [27]	50	Telea [45]	23.39±8.17	2.597±0.062	<1
DIBR	MiDaS-L [27]	75	Telea [45]	22.34±8.25	4.327±0.127	<1
DIBR	DD-S [26]	25	Fast	31.01±9.23	0.400±0.017	∼3
DIBR	DD-S [26]	50	Fast	24.61±6.34	0.409±0.021	∼2
DIBR	DD-S [26]	75	Fast	26.15±6.51	0.406±0.025	∼2
DIBR	DD-S [26]	25	NS [46]	23.91±6.63	0.649±0.130	∼2
DIBR	DD-S [26]	50	NS [46]	24.65±6.35	1.095±0.231	<1
DIBR	DD-S [26]	75	NS [46]	26.22±6.53	1.906±0.478	<1
DIBR	DD-S [26]	25	Telea [45]	23.90±6.63	1.990±0.696	<1
DIBR	DD-S [26]	50	Telea [45]	24.64±6.35	5.347±1.438	<1
DIBR	DD-S [26]	75	Telea [45]	26.20±6.53	9.381±2.471	<1
DIBR	DD [25]	25	Fast	24.19±6.77	0.726±0.022	∼1
DIBR	DD [25]	50	Fast	24.44±6.12	0.734±0.016	∼1
DIBR	DD [25]	75	Fast	25.71±6.06	0.773±0.010	∼1
DIBR	DD [25]	25	NS [46]	31.12±9.29	1.218±0.296	<1
DIBR	DD [25]	50	NS [46]	24.65±6.35	2.230±0.875	<1
DIBR	DD [25]	75	NS [46]	26.22±6.53	3.635±1.739	<1
DIBR	DD [25]	25	Telea [45]	31.09±9.28	2.773±1.002	<1
DIBR	DD [25]	50	Telea [45]	24.64±6.35	6.506±2.921	<1
DIBR	DD [25]	75	Telea [45]	26.20±6.53	10.736±4.602	<1
Deep3D [37]	–	–	–	22.69±6.79	0.032±0.004	∼31

**Table 3:** The results of the qualitative (user) study averaged across the 5 study participants. Each participant in the experiment watched each of the 92 videos in random order, rating each video on a scale of 1 to 10 (according to whether they had a 3D experience and whether they felt comfortable watching the video). A rating of 10 meant complete approval.

Method	‘Do You Perceive 3D?’	‘Do You Feel Comfortable of Viewing the Stereo Panoramas?’
Deep3D [37]	3.34±2.14	8.47±1.59
MiDaS, FAST [27], MaxDisp. = 25	3.72±2.37	8.40±1.57
MiDaS, FAST [27], MaxDisp. = 50	6.44±2.83	7.45±1.65
MiDaS, FAST [27], MaxDisp. = 75	6.76±2.69	7.17±1.55

## Data Availability

Source codes can be downloaded from: https://github.com/browarsoftware/stereofast, accessed on 16 June 2023.
